# A Comparative Study on the Expression Levels of the HMEI Gene Family in *Hirudinaria manillensis* and *Whitmania pigra*

**DOI:** 10.3390/biology15211973

**Published:** 2026-11-02

**Authors:** Pinpin Zhou, Yanping Pan, Ming Yang, Yiyong Luo, Huanhuan Chen, Zichao Liu, Fang Zhao, Lizhou Tang, Gonghua Lin

**Affiliations:** 1 Key Laboratory of Biodiversity Conservation and Bioresource Utilization of Jiangxi Province, College of Life Sciences, Jiangxi Normal University, Nanchang 330022, China; zhou166833@outlook.com (P.Z.); 15581461344@163.com (Y.P.); yyongl-168@163.com (Y.L.); 2 Cancer Center, Laboratory of Oncogene, State Key Laboratory of Biotherapy, West China Hospital, Sichuan University, Chengdu 610041, China; qjyangming@163.com; 3 College of Biology and Food Engineering, Qujing Normal University, Qujing 655011, China; chhuanhuan@163.com; 4 Yunnan International Joint Laboratory with South and Southeast Asia for the Integrated Development of Animal-Derived Anti-Thrombosis Chinese Medicine, Qujing Normal University, Qujing 655011, China; 5 School of Agriculture and Life Sciences, Kunming University, Kunming 650214, China; abclzc@aliyun.com; 6 Key Laboratory of Jiangxi Province for Functional Biology and Pollution Control in Red Soil Regions, School of Life Sciences, Jinggangshan University, Ji’an 343009, China; zhaofang@jgsu.edu.cn

**Keywords:** *Hirudinaria manillensis*, *Whitmania pigra*, HMEI gene family, anti-inflammation, differential gene expression, elastase inhibitor

## Abstract

Leeches are classic animal-derived traditional Chinese medicines for activating blood circulation and eliminating blood stasis. The hematophagous *Hirudinaria manillensis* and the non-hematophagous *Whitmania pigra* are the two most widely used medicinal leeches, but their HMEI (*H. manillensis* elastase inhibitor) gene family, a gene family containing candidate elastase-inhibitor paralogs, has not been systematically compared across species and geographical populations. Here, we performed RNA-Seq on head tissues from five populations of each species and compared the expression of all HMEI paralogs. The 38 paralogs formed 9 descriptive sequence groups. Total HMEI expression showed no significant interspecies difference, yet the two species showed distinct transcriptional patterns in the sampled populations: expression in *H. manillensis* was concentrated in a few paralogs with ultra-high abundance, whereas expression in *W. pigra* was distributed across multiple moderately expressed paralogs. Total HMEI expression did not differ significantly among *H. manillensis* populations, whereas significant inter-population differences were detected in *W. pigra*. Four expression-based candidates for functional validation were identified: *HMEI_Hman06* and *HMEI_Hman15* from *H. manillensis*, and *HMEI_Wpig01* and *HMEI_Wpig17* from *W. pigra*. These results characterize interspecies and population-associated variation in HMEI expression, providing a transcriptomic resource for comparative biochemical and functional characterization of HMEI paralogs.

## Simple Summary

Leeches contain diverse bioactive molecules, but the functions of many genes remain unclear. We compared the expression of the HMEI (*Hirudinaria manillensis* elastase inhibitor) gene family in the head tissues of 54 *Hirudinaria manillensis* and 57 *Whitmania pigra* individuals collected from 5 geographical populations per species. Although total HMEI transcript abundance did not differ significantly between the two species, the distribution of expression among individual paralogs differed. Among the reliably quantified paralogs, *HMEI_Hman06* and *HMEI_Hman15* in *H. manillensis* and *HMEI_Wpig01* and *HMEI_Wpig17* in *W. pigra* showed relatively high expression. Several paralogs also showed population-level expression differences. The results identify expression-based candidates for future studies of HMEI biochemical properties and potential anti-inflammatory activity.

## 1. Introduction

Leeches, including both hematophagous and non-hematophagous species, are annelids belonging to the class Hirudinea. They are widely distributed in freshwater environments such as rivers, lakes and rice paddies. Approximately 600 leech species have been recorded globally, and over 100 species have been identified in China, covering genera including *Hirudinaria*, *Whitmania* and *Haemopis* [1,2,3]. Leeches possess prominent pharmacological activities including anticoagulation, thrombolysis and anti-inflammation, so the development and utilization of their bioactive components have attracted sustained research attention [4,5]. Among medicinally relevant leech species, *Hirudinaria manillensis* and *Whitmania pigra* represent important research models in China [6,7]. *H. manillensis* belongs to the genus *Hirudinaria*, family Hirudinidae [8]. It is distributed across China and Southeast Asian countries including the Philippines and Malaysia, with core habitats in southern Guangxi and Guangdong Provinces of China [6,9]. As an obligate hematophage feeding on the blood of buffalo and other mammals [6,10], this leech features large body size, fast growth rate and strong biological activity [11,12]. It is included in the regional herbal medicine standards of Guangxi [13] and Yunnan [14] as an essential antithrombotic animal medicinal material, and circulates in the market as dried whole bodies or lyophilized powder herbal slices [13,14]. *W. pigra* is classified into the genus *Whitmania*, family Hirudinidae, with a broader distribution range across freshwater habitats in North, East and Central China [15]. It feeds on snail body fluids and plankton [16,17]. As a non-hematophagous medicinal leech, it exerts therapeutic effects of breaking blood stagnation, unblocking meridians, dissipating stagnation masses and eliminating swelling. It is specified in Volume I of the 2025 Edition of the Pharmacopoeia of the People’s Republic of China (PPRC) and is the most commonly used leech material in clinical practice [18]. Divergent feeding habits lead to distinct profiles of bioactive components and separate clinical application scenarios, making the two leeches ideal comparative models for mining therapeutic gene resources.

Hirudin is the classic bioactive component of leeches that specifically inhibits thrombin. Several modified hirudin derivatives (e.g., lepirudin, desirudin and bivalirudin) have been applied in clinical anticoagulation treatment [19,20]. With the advancement of genomics and transcriptomics, genome assembly and salivary transcriptome analyses of the European medicinal leech *H. medicinalis* and its close relatives *H. verbana* and *H. orientalis* have revealed a broad repertoire of anticoagulant genes [21,22], and transcriptomic studies of *H. manillensis* have further enriched the expression profiles of such genes in this species [23]. Researchers have identified multiple antithrombotic gene families in *H. manillensis* and *W. pigra*, such as hirudin, Lefaxin, Antistasin, LDTI and Destabilase [6,7,16]. These families act on different targets of hemostasis and inflammation, including thrombin targeted by hirudin, coagulation factor Xa targeted by Lefaxin and Antistasin, mast cell tryptase targeted by LDTI, and fibrin targeted by Destabilase; together, they constitute a multi-target antithrombotic regulatory network [7,24,25,26,27]. Evolutionary differences in feeding habits reshape antithrombotic gene families [7,28,29,30,31,32]. Comparative genomic analyses of multiple non-hematophagous leeches have provided direct evidence that contraction or loss of anticoagulant gene families accompanies the transition from blood-feeding to non-blood-feeding lifestyles [33]. The hirudin family undergoes gene expansion and high salivary expression in hematophagous *H. manillensis*, endowing this species with strong overall anticoagulant activity. In contrast, functional characterization of the putative hirudins of non-hematophagous *W. pigra* showed that some variants inhibit thrombin, whereas others (hirudin-like factors) lack or show only very weak thrombin-inhibitory activity; the molecular basis of these functional differences and the biological targets of the non-inhibitory variants remain to be characterized [17]. Peptidomics, proteomics and metabolomics continuously identify novel short anticoagulant peptides from leech extracts, and comparative analyses of the anticoagulant components of different leech species have provided initial clues to species-specific bioactive profiles [34,35,36,37]. Proprietary Chinese medicines containing leech extracts are widely used to treat ischemic cardiovascular and cerebrovascular diseases [38]. To date, most leech studies focus on anticoagulation and thrombolysis, while investigations on leech anti-inflammatory activity remain insufficient. Inflammation is an essential defensive response of organisms to tissue injury and pathogen invasion, yet excessive or persistent inflammation induces a wide range of human diseases [39,40,41]. In addition, in the context of leech research, few studies elaborate the inflammatory pathological pathways linking inflammation and thrombosis [42,43], which prevents comprehensive interpretation of the molecular mechanisms behind leeches’ blood-activating and stasis-resolving effects. Therefore, exploring the anti-inflammatory mechanisms of leeches is a critical scientific task.

HMEI is the abbreviation for *Hirudinaria manillensis* elastase inhibitor. It was named after its initial isolation and identification from *H. manillensis*, and is considered a key bioactive component implicated in leech anti-inflammatory effects [44]. Previous genomic sequencing revealed that the *H. manillensis* genome contains at least 72 antithrombotic genes belonging to 21 gene families. The HMEI family is the largest among these families, containing 18 paralogs [6]. Each HMEI protein is predicted to form a rigid disulfide-bond binding pocket via 10 conserved cysteine residues, which is proposed to bind the active center of neutrophil elastase (NE) [6]. However, to date only HMEI-A of *H. manillensis* has been functionally characterized. HMEI-A was verified to exert potent elastase inhibitory activity with an inhibition constant (Ki) of 1.69 × 10^−8^ mol·L^−1^ and no obvious inhibitory effects on trypsin, thrombin or other proteases [44]. HMEI-B shares high sequence homology with HMEI-A, especially in conserved cysteine residues; hence it is hypothesized to have similar biochemical properties, although this remains experimentally unvalidated [44]. Importantly, because the amino acid sequence identity among the HMEI paralogs ranges from approximately 53% to 99% [6], different paralogs may have entirely different biological functions, and the elastase-inhibitory activities of the remaining paralogs remain to be experimentally determined. Two anti-inflammatory mechanisms of HMEI have been hypothesized. First, by inhibiting NE, HMEI may suppress NE-triggered vascular endothelial injury, tissue edema and local inflammatory infiltration [44,45]. Second, HMEI may block NE-mediated NF-κB and NLRP3 pyroptosis pathways, downregulating the secretion of pro-inflammatory factors including IL-1β, IL-18 and C-reactive protein (CRP), thereby relieving chronic vascular inflammation and lipid deposition on vascular walls [45]. NE is also a core structural protein of neutrophil extracellular traps (NETs); by inhibiting NE and reducing NET release, HMEI may break the vicious cycle between inflammation and thrombosis [42,43].

In addition to the HMEI family of *H. manillensis* described above, we previously identified 20 HMEI paralogs in the genome of the non-hematophagous *W. pigra* [16], indicating that the HMEI family is widely distributed across leech species and may represent a valuable resource for cross-species pharmaceutical development. We also compared the overall transcriptional abundance of HMEI between *H. manillensis* and *W. pigra* and found no significant interspecies difference in total HMEI expression [16]. However, a systematic paralog-level comparison of HMEI expression between the two species has not been conducted. In the present study, we therefore compared the expression levels of individual HMEI paralogs between the two medicinal leeches using RNA-Seq data and characterized the evolutionary and transcriptional differentiation patterns of this gene family. This work provides transcriptomic evidence for prioritizing candidate genes with high transcriptional abundance and for generating testable hypotheses on the divergent bioactive profiles of the two leeches, thereby supporting future functional validation and the development of leech-derived bioactive lead molecules.

## 2. Materials and Methods

### 2.1. RNA Sequencing

**Figure 1:**
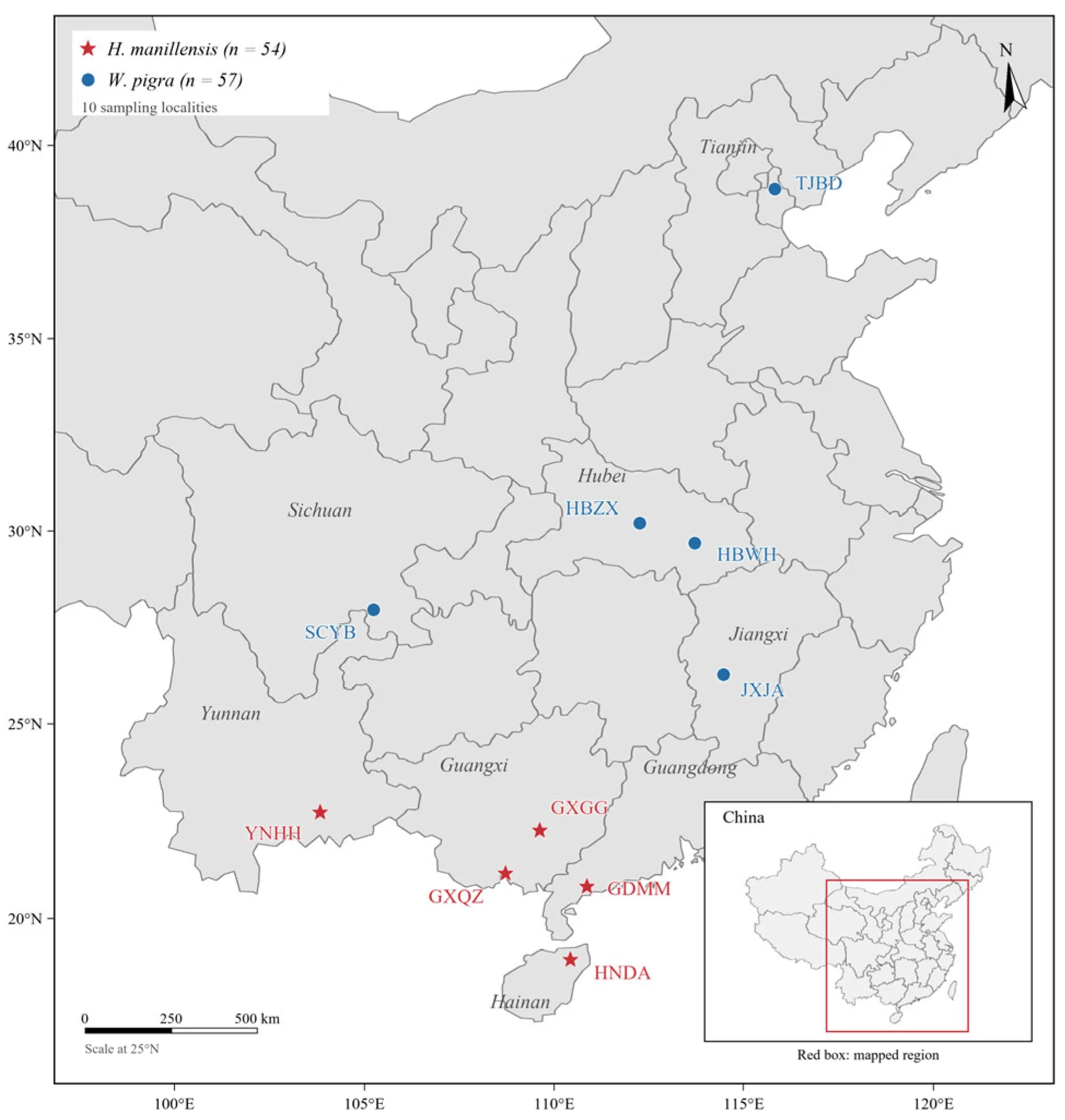
Sampling locations of *H. manillensis* and *W. pigra* in China. Symbols identify species; locality codes correspond to Table 1.

All leech samples were live-captured in China, with GPS coordinates recorded for each sampling site. The sampling localities are shown in Figure 1, and the detailed sampling information—including locality codes, geographic coordinates and sample sizes for each population—is summarized in Table 1. All specimens were identified based on morphological examination in the field, and their species identities were further confirmed by mitochondrial gene sequence analysis, as validated in our population genetic study [6,9,16,46]. The collected leeches were kept static in water tanks covered with 80-mesh nets for two weeks, and then their head tissues were dissected, and no pooling of tissue or RNA was performed at any stage.

Total DNA was extracted from head tissues using the DNeasy Blood and Tissue Kit (Qiagen, Hilden, Germany), and total RNA was isolated from head tissues with TRIzol Reagent (Invitrogen, Carlsbad, CA, USA) and purified using the RNeasy Mini Kit (Qiagen, Hilden, Germany). cDNA libraries with an average insert size of 350 bp were constructed using intact RNA samples compatible with Illumina sequencing platforms. RNA sequencing was carried out on the BGISeq platform. Raw sequencing reads were quality-filtered with fastp v0.20.0 [47] to obtain high-quality clean reads for subsequent analysis.

### 2.2. Phylogenetic Analysis

To evaluate whether all HMEI paralogs represent functional genes, the coding sequence of each paralog (File S1) was examined for the presence of premature stop codons and the completeness of the open reading frame (File S2). For *HMEI_Wpig07*, which contains a single-base frameshift, an ambiguous base (N) was inserted to restore the reading frame prior to this step. The CDS nucleotide sequences of all 18 *H. manillensis* and 20 *W. pigra* HMEI paralogs were then translated into protein sequences, aligned using ClustalW implemented in MEGA v11 [48], and back-translated to the nucleotide alignment for phylogenetic analysis. Phylogenetic reconstruction was carried out with the maximum likelihood (ML) method under the Tamura–Nei (TN93) substitution model with gamma-distributed rate heterogeneity (+G, five categories) and a proportion of invariant sites (+I), with gaps and missing data treated by partial deletion (95% site coverage cutoff). The initial tree was obtained by neighbor-joining and BioNJ, and the ML tree was optimized using the nearest-neighbor interchange (NNI) heuristic. Branch support was assessed with 1000 bootstrap replicates. The resulting ML tree was displayed as an unrooted phylogram and was further processed using FigTree v1.4.4 for presentation, with branch lengths and bootstrap support values retained. The amino acid sequences of all HMEI paralogs from both species are provided in File S3, and the multiple nucleotide sequence alignment used for phylogenetic tree reconstruction is provided in File S4.

### 2.3. Expression Analysis of the HMEI Gene Family

We used all coding region sequences annotated from whole-genome structural data (File S1) as templates to build sequence indexes with the Salmon software v1.0.0 [49]. Transcriptomic reads of each sample were mapped to the indexes with a *k*-mer value of 31. The keepDuplicates option of Salmon was enabled to retain reads that map equally well to multiple paralogs, including identical or nearly identical copies. We calculated TPM (transcripts per million) values for each coding sequence (provided in File S5) to quantify relative gene expression levels. The sum of TPM values of all HMEI in one sample was defined as the total HMEI expression of that sample. The unique and ambiguous read counts for all HMEI paralogs were extracted from the Salmon output (aux_info/ambig_info.tsv) (File S6). Pairwise nucleotide distances among all HMEI paralogs were calculated from the codon-aware alignment used for tree reconstruction to assess potential ambiguity in transcript quantification (File S7). For the paralogs with identical nucleotide sequences (*HMEI_Hman03/04/05* and *HMEI_Wpig12/13*), genomic coordinates were retrieved from the respective genome annotations (File S8), confirming that these copies correspond to distinct, non-overlapping genomic loci.

We adopted the Mann–Whitney *U* test to compare total HMEI expression between *H. manillensis* and *W. pigra*. To ensure statistical validity, we first performed the Shapiro–Wilk test to detect the normality of expression data. Since the data failed normality tests, we applied the nonparametric Mann–Whitney *U* test to guarantee reliable analytical results. The Mann–Whitney *U* test was performed using the untransformed per-sample total HMEI TPM values. For visualization in the violin plot, the total HMEI TPM value of each individual was transformed as log2(TPM + 1). Kernel density estimation was restricted to the observed data range to prevent the violin outline from extending below zero.

### 2.4. Statistical Analyses of Inter-Population Expression

We pooled expression data of all HMEI from different geographical populations of *H. manillensis* and *W. pigra* and calculated the mean ± standard error (mean ± SE) of each gene within each population using SPSS 26.0 (IBM Corp., Armonk, NY, USA) [50]. The Shapiro–Wilk test and Levene’s test were used to assess the normality and homogeneity of variance of the data; because these assumptions were not met, the nonparametric Kruskal–Wallis *H* test was applied to detect significant inter-population expression differences for each gene, and *P* values were corrected for multiple testing across paralogs using the Benjamini–Hochberg false discovery rate (FDR) procedure separately within each species (adjusted *p* < 0.05 was considered statistically significant).

Clustering heatmaps were generated with Origin 2021 (OriginLab Corporation, Northampton, MA, USA) [51]. Before visualization, TPM values were transformed as log2(TPM + 1) to reduce the influence of highly expressed paralogs and improve visualization of low-expression differences. Heatmap colours directly represent the log2(TPM + 1) values, and no further normalization was applied, consistent with the heatmap presentation used in comparable gene-family expression studies [52,53]. Hierarchical clustering was performed using Euclidean distance and average linkage. To test whether total HMEI expression differed among populations within each species, the Kruskal–Wallis *H* test was applied to the per-sample total TPM values within each species, followed by Dunn’s post hoc tests with Benjamini–Hochberg correction.

### 2.5. Inter-Gene Expression Analysis

We analyzed transcriptome-derived relative expression data via SPSS 26.0 [50] to calculate the mean ± SE of each gene, following the same workflow as inter-population expression analysis. The Friedman test was then applied to detect significant expression differences among individual paralogs within the HMEI gene family of the two leech species. Pairwise differences among paralogs were assessed using Wilcoxon signed-rank tests, and the resulting *p* values were corrected using the FDR procedure (File S9). We generated bar charts with GraphPad Prism 10.1.2 (GraphPad Software, San Diego, CA, USA) [54] based on TPM-normalized expression data to visually display expression differences of each HMEI paralog in *H. manillensis* and *W. pigra*.

## 3. Results

### 3.1. Phylogenetic Relationships and Group-Level Expression of HMEI

**Figure 2:**
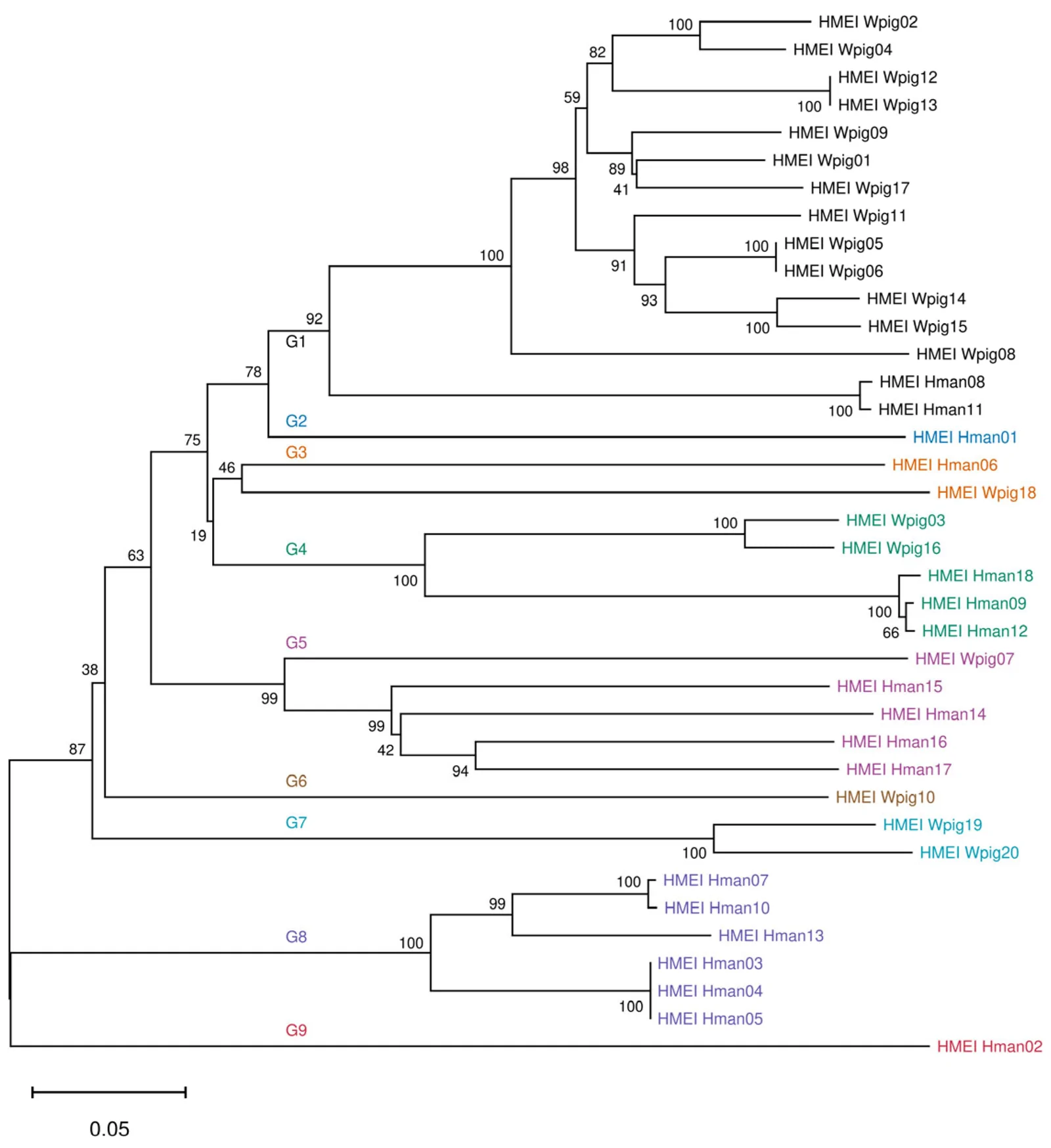
Unrooted maximum-likelihood phylogram of the HMEI gene family in *H. manillensis* and *W. pigra*. The tree includes 18 HMEI sequences from *H. manillensis* and 20 from *W. pigra*. Gene labels are coloured to indicate the nine descriptive sequence groups (G1–G9). Bootstrap support values are shown at nodes. Branch lengths represent estimated substitutions per site; the scale bar indicates 0.05 substitutions per site.

The phylogenetic tree (Figure 2) revealed interleaved clustering of HMEI from *H. manillensis* and *W. pigra*. All HMEI sequences were assigned to nine descriptive sequence groups (Table 2), designated G1–G9, based on the clustering pattern in the unrooted maximum-likelihood tree. These groups are used to summarize sequence relationships and expression patterns. G1/3/4/5 contain paralogs from both species, whereas G2/8/9 are exclusive to *H. manillensis* and G6/7 only contain *W. pigra* sequences. G1 is the largest group, dominated by thirteen *W. pigra* paralogs with a small number of *H. manillensis* genes are *HMEI_Hman08/11*. G3/4/5 also contain paralogs from both species: G3 consists of *HMEI_Hman06* and *HMEI_Wpig18*, G4 consists of *HMEI_Wpig03/16*, *HMEI_Hman18/09/12*, and G5 consists of *HMEI_Wpig07*, *HMEI_Hman15/14/16/17*. G2, G8 and G9 are exclusive to *H. manillensis*: G8 comprises *HMEI_Hman04/05/03/13/07/10*, whereas G2 and G9 are single-member groups containing *HMEI_Hman01/02*, respectively. G6 and G7 only contain *W. pigra* sequences: G7 consists of *HMEI_Wpig19/20*, and G6 is a single-member group containing *HMEI_Wpig10*. Most terminal branches had bootstrap values ≥80%, indicating stable clustering topology. It should be noted that G3, consisting of *HMEI_Hman06* and *HMEI_Wpig18*, was the only group with 46 bootstrap support. This group was therefore treated as an unresolved group, and the two paralogs were provisionally retained as a cluster solely on the basis of the displayed topology, from which no evolutionary inference was drawn. Therefore, G1–G9 were used as descriptive sequence groups based on the displayed clustering topology and the support of their corresponding stem branches, rather than as definitive evolutionary lineages. Low support values at some deep nodes reflect uncertainties about early evolutionary differentiation events.

We compared the average TPM expression of each descriptive sequence group between the two species. G3 showed the highest expression in *H. manillensis*, driven mainly by *HMEI_Hman06*, whereas its *W. pigra* member *HMEI_Wpig18* showed moderate expression. G5 exhibited high expression in *H. manillensis* but low expression in *W. pigra*. G4 showed comparable moderate expression in both species. G1 showed much higher expression in *W. pigra* than in *H. manillensis*. Intraspecies comparison further showed distinct expression hierarchies. In *H. manillensis*, expression levels decreased sequentially from G3 to G5 and G4, while G2, G8, G9 and G1 presented low or negligible expression. In *W. pigra*, G3 and G1 showed comparable moderate-to-high expression, followed by gradually reduced expression in G5, G4 and G7. The remaining groups had negligible transcription signals. Expression levels varied greatly across groups, demonstrating non-corresponding differential expression of HMEI between the two species.

We selected the highest-expressed paralog from each group as the representative gene for cross-species comparison (Table 3). For *H. manillensis*, *HMEI_Hman06* had the highest expression, followed by *HMEI_Hman15* and *HMEI_Hman09*. For *W. pigra*, *HMEI_Wpig01* showed the maximum expression, with *HMEI_Wpig18* and *HMEI_Wpig16* ranking second and third. The relative expression of the representative paralogs varied between groups without a consistent species-level direction. In G1, the *W. pigra* representative *HMEI_Wpig01* was expressed far more highly than its *H. manillensis* counterpart *HMEI_Hman08*. In G3, G4 and G5, by contrast, the *H. manillensis* representatives exceeded their *W. pigra* counterparts, most markedly in G3, in which *HMEI_Hman06* reached the highest values among all representatives, whereas the difference was modest in G4. Representatives of the species-specific groups were all lowly expressed, ranging from nearly undetectable levels to moderate values.

### 3.2. Inter-Species Comparison of Total HMEI Expression

Shapiro–Wilk normality tests showed that expression data of both *H. manillensis* (*p* < 0.001) and *W. pigra* (*p* < 0.001) violated normal distribution. The nonparametric Mann–Whitney *U* test indicated no statistically significant difference in total HMEI expression between the two species (*p* = 0.271).

**Figure 3:**
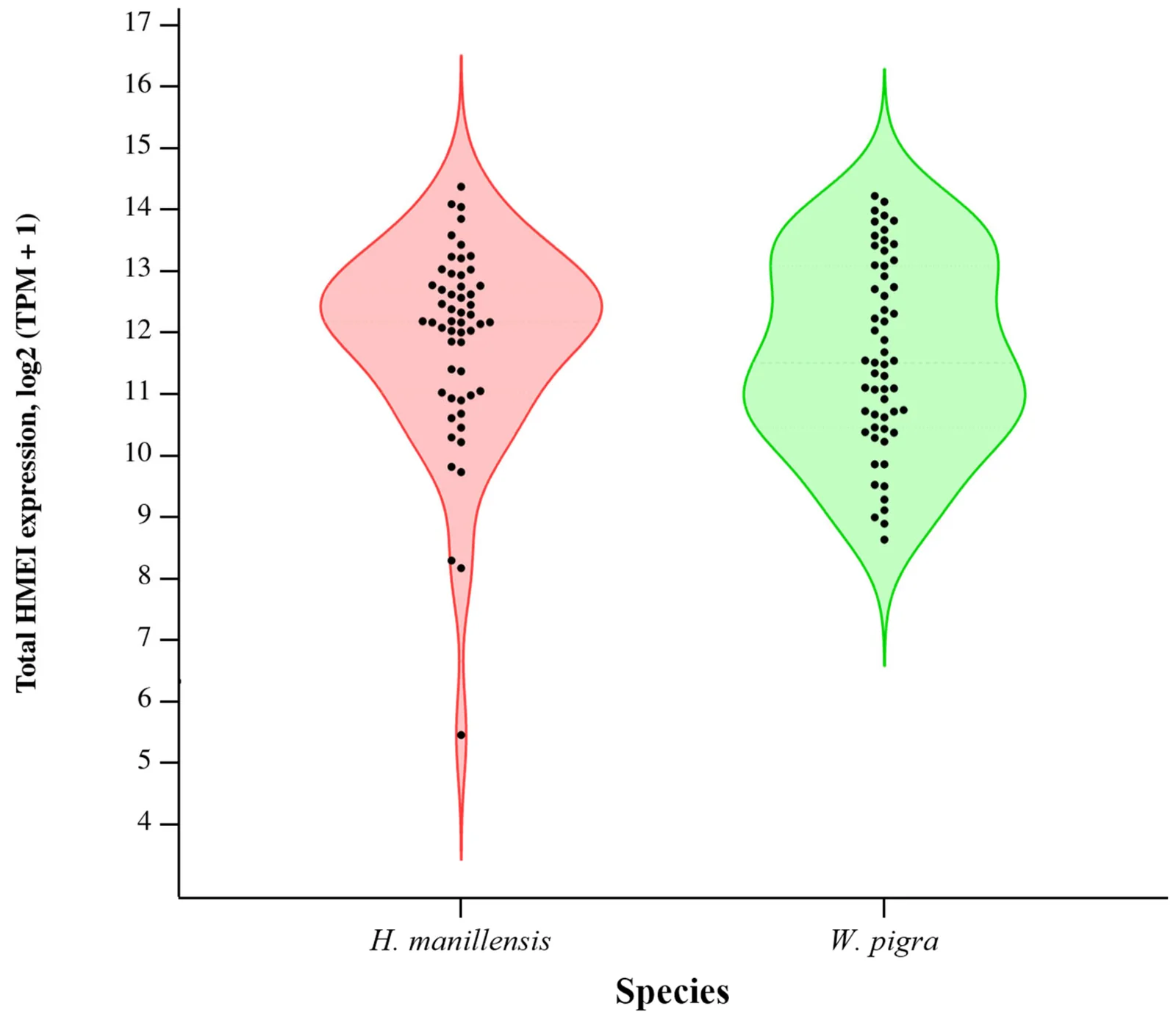
Violin plot showing the distribution and dispersion of HMEI expression in individual samples of *H. manillensis* and *W. pigra*. Total HMEI TPM values were visualized after log2(TPM + 1) transformation, and violin densities were restricted to the observed data range. The width of each violin outline represents kernel density of samples at corresponding expression levels, and internal black dots stand for total expression values of individual biological replicates. The inter-species difference was tested by the Mann–Whitney *U* test (*p* = 0.271, not significant) (*n* = 54 for *H. manillensis*; *n* = 57 for *W. pigra*).

The violin plot (Figure 3) illustrates the distribution and dispersion of the per-sample total HMEI TPM values in the two species. Total HMEI expression was right-skewed in both species, with most samples clustered within low-expression ranges and only a small subset of outlier samples showing extremely high expression. The median total HMEI TPM was 4626.58 (IQR: 2064.07–7200.75) in *H. manillensis* and 2920.40 (IQR: 1392.47–8731.30) in *W. pigra*. Relative to *W. pigra*, *H. manillensis* showed a higher median value and a larger proportion of samples with medium-to-high expression, whereas *W. pigra* samples showed lower overall expression but a wider between-sample dispersion (IQR). Despite these distributional differences, the Mann–Whitney *U* test confirmed no significant interspecies difference in total HMEI expression (*p* = 0.271). These results indicate that the difference in HMEI expression between the two species lies in the distribution of transcript abundance among individual paralogs rather than in overall transcript abundance.

### 3.3. Inter-Population Expression Analysis

#### 3.3.1. Inter-Population Expression of *Hirudinaria manillensis*

SPSS analytical results (Table 4) showed that the HNDA population had the highest mean total HMEI expression and the GXGG population the lowest among the five populations. However, the Kruskal–Wallis *H* test on per-sample total HMEI TPM values detected no significant difference among the five populations (*H* = 7.765, *df* = 4, *p* = 0.101). Per-gene Kruskal–Wallis *H* tests (with Benjamini–Hochberg FDR correction, File S9) detected significant inter-population expression differences for 9 paralogs (01, 02, 08, 09, 11, 15, 16, 17, 18; *q* < 0.05); the remaining 9 paralogs showed no obvious population divergence.

**Figure 4:**
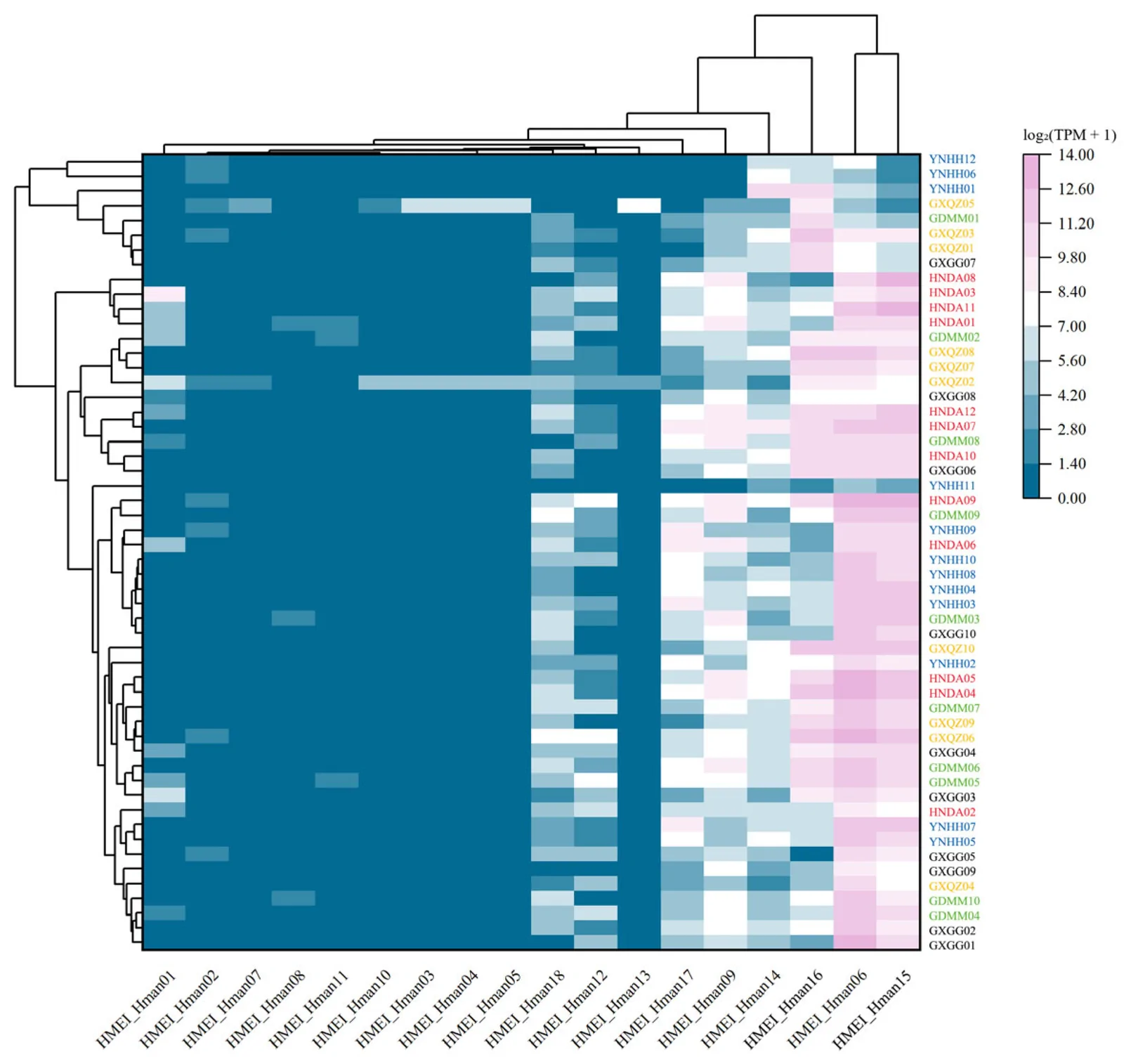
Clustering heatmap of expression of HMEI gene family members across different populations of *H. manillensis*. Rows correspond to individual samples from distinct populations, while columns represent each HMEI gene. Color gradients indicate gene expression, with pink representing high gene expression and blue representing low gene expression. Heatmap values are log2 (TPM + 1)-transformed TPM values, and no further normalization was applied; the colour scale (0–14) directly represents these values. The dendrograms reflect the similarity in gene expression patterns among individual samples. Each row represents one individual biological replicate; *n* = 10–12 per population (Table 1). Population labels are colour-coded by geographical origin.

The clustering heatmap (Figure 4) showed that the expression pattern of HMEI paralogs in *H. manillensis* was largely consistent among populations, with no obvious population-specific expression profile. *HMEI_Hman06* and *HMEI_Hman15* were persistently and highly expressed among reliably quantifiable paralogs across all sampled populations, whereas the remaining paralogs showed extremely low or nearly undetectable transcription in every population. Although a few paralogs showed statistically significant inter-population differences (File S9), the overall pattern was dominated by the persistently high expression of *HMEI_Hman06* and *HMEI_Hman15*. Accordingly, *HMEI_Hman06* and *HMEI_Hman15* represent stable high-expression candidate genes of *H. manillensis* for subsequent anti-inflammatory functional validation. These data provide guidance for screening representative HMEI and dissecting their anti-inflammatory molecular mechanisms.

#### 3.3.2. Inter-Population Expression of *Whitmania pigra*

SPSS analysis (Table 5) showed that the JXJA population had the highest mean total HMEI expression and the HBZX population the lowest among the five populations. The Kruskal–Wallis *H* test on per-sample total HMEI TPM values detected a significant difference among populations (*H* = 16.291, *df* = 4, *p* = 0.003), and Dunn’s post hoc tests (Benjamini–Hochberg corrected) showed that JXJA was significantly higher than HBZX (*Padj* = 0.0006); the remaining pairwise comparisons were not significant after correction. Per-gene Kruskal–Wallis *H* tests (with Benjamini–Hochberg FDR correction, File S9) revealed 3 paralogs (08, 10, 20) with no significant inter-population expression differences; the other 17 paralogs exhibited obvious population divergence.

**Figure 5:**
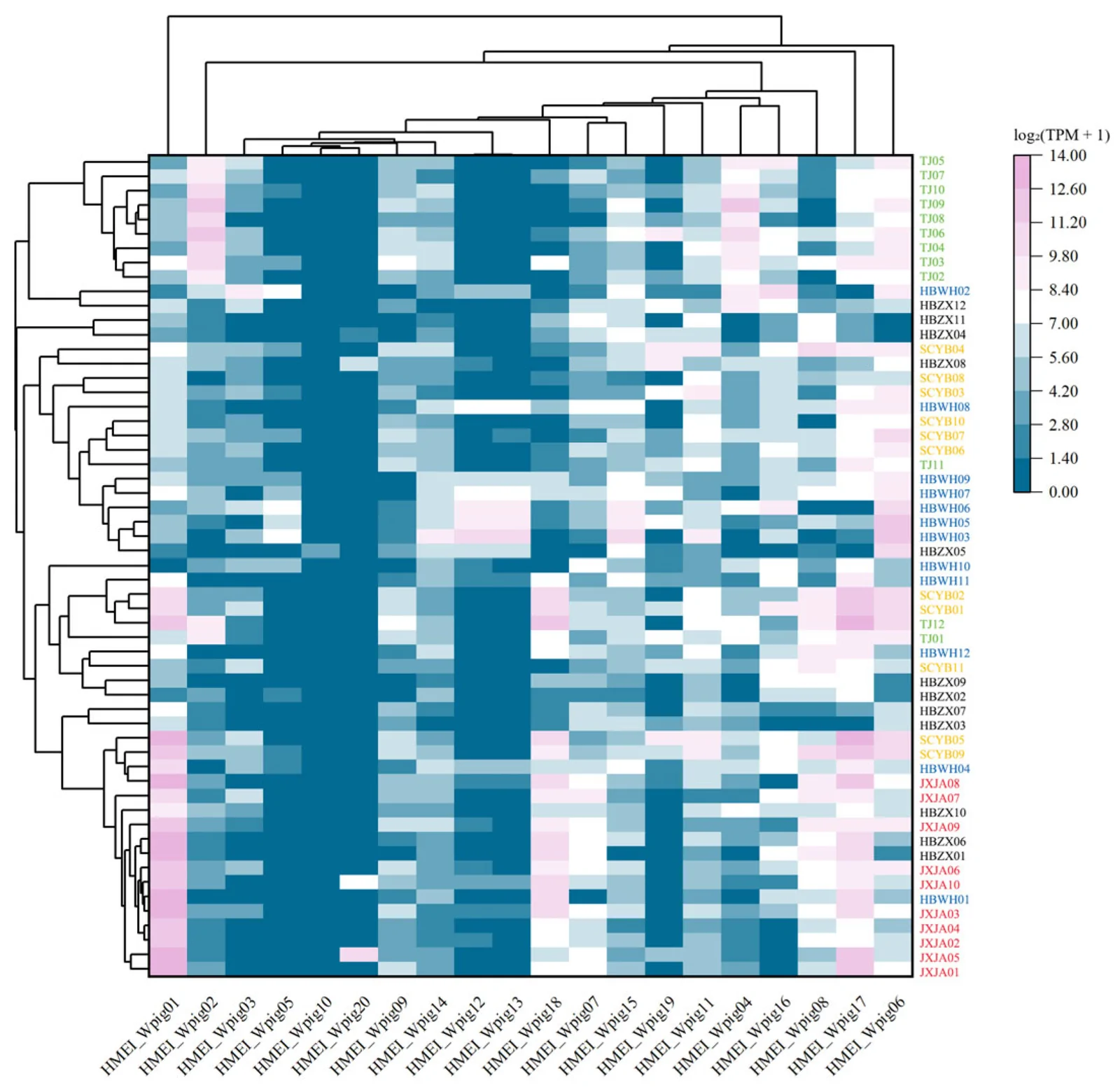
Clustering heatmap of HMEI gene member expression across distinct populations of *W. pigra*. Rows correspond to individual samples from distinct populations, while columns represent each HMEI gene. Color gradients indicate gene expression, with pink representing high gene expression and blue representing low gene expression. Heatmap values are log2 (TPM + 1)-transformed TPM values, and no further normalization was applied; the colour scale (0–14) directly represents these values. The dendrograms reflect the similarity in gene expression patterns among individual samples. Each row represents one individual biological replicate; *n* = 10–12 per population (Table 1). Population labels are colour-coded by geographical origin.

The clustering heatmap (Figure 5) showed pronounced inter-population differences in the expression profiles of HMEI paralogs in *W. pigra*. In contrast to *H. manillensis*, where only a few paralogs were expressed at extremely high levels and the remaining paralogs were barely detectable, the *W. pigra* HMEI family exhibited a more dispersed expression profile. Most paralogs were expressed at low-to-moderate levels across multiple populations, and only a few paralogs reached high expression in specific populations. *HMEI_Wpig01* was extremely enriched in JXJA, *HMEI_Wpig01* was highly enriched in HBZX, *HMEI_Wpig02* in TJBD, *HMEI_Wpig17/01* in SCYB and *HMEI_Wpig06* in HBWH. Consequently, each geographical population was characterized by a distinct set of moderately-to-highly expressed HMEI paralogs, whereas the *H. manillensis* profile was dominated by the high expression of a few paralogs with negligible expression of the remainder. This contrast is critical for comparing the divergent transcriptional patterns of the HMEI family between the two species and for investigating population-associated expression variation in *W. pigra*.

### 3.4. Inter-Paralog Expression Analysis

In the following sections, expression levels of individual paralogs are compared within each species (paralog-level, within-species comparison) using the Friedman test, in contrast to the group-level, cross-species comparison presented in Section 3.1 (Table 2). In *HmanGDMM01* and *WpigHBWH01*, used as examples for the respective species, most reads assigned to the highly expressed paralogs *HMEI_Hman06*, *HMEI_Hman15*, *HMEI_Wpig01*, and *HMEI_Wpig17* were uniquely mapped (≥88%; File S6). Each paralog also showed clear sequence divergence from its nearest paralog (*p*-distance = 0.09–0.40; File S7), supporting the reliability of their TPM estimates. In contrast, the reads of *HMEI_Hman16* and *HMEI_Wpig06* were almost entirely ambiguous, their TPM values are therefore not interpreted as independent quantitative evidence. Within the remainder of the family, the identical-sequence paralogs *HMEI_Hman03/04/05* and *HMEI_Wpig12/13* were confirmed to occupy independent genomic loci (File S8) and were retained as separate paralogs in the testing.

#### 3.4.1. Inter-Paralog Expression of *Hirudinaria manillensis*

**Figure 6:**
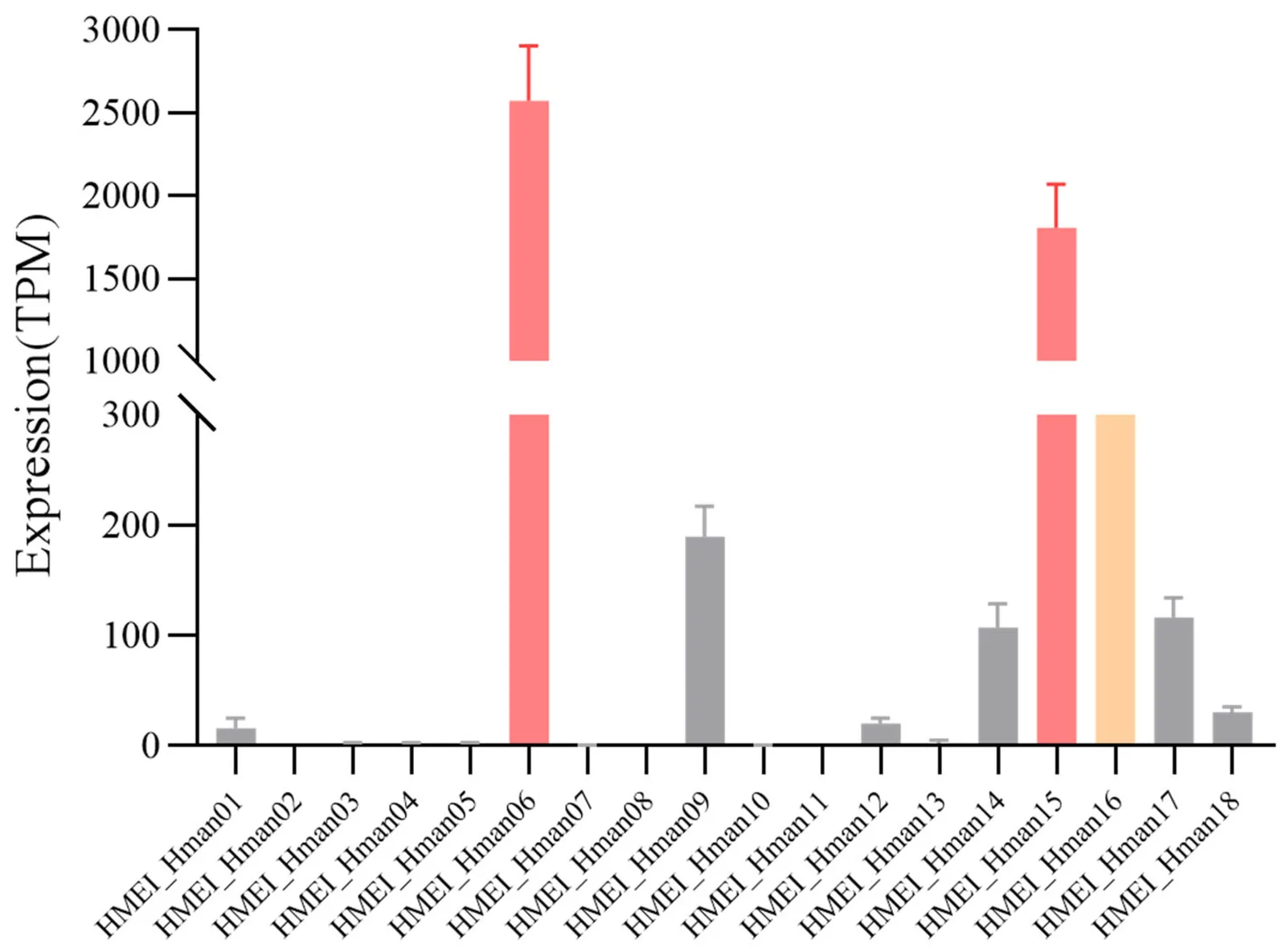
Expression levels of individual genes in the HMEI gene family of *H. manillensis*. The vertical axis indicates gene expression levels; the horizontal axis indicates each HMEI gene. Bar heights correspond to mean expression values. Bars are colour-coded by mean TPM: red, mean TPM ≥ 1000; yellow, 500 ≤ mean TPM < 1000; grey, mean TPM < 500. Error bars denote the standard error across biological replicates, and a broken *y*-axis was applied to accommodate the wide range of transcriptional abundance. Data are based on *n* = 54 biological replicates.

SPSS results (Table 6) and Friedman test indicated extremely significant expression differences among the 18 HMEI of *H. manillensis* (*χ^2^* = 737.045, *df* = 17, *p* < 0.001). The bar chart (Figure 6) intuitively showed three highly expressed paralogs (06, 15, 16) in the HMEI gene family of *H. manillensis*. *HMEI_Hman06* had the highest overall transcriptional abundance across all paralogs, followed by *HMEI_Hman15*. Their expression levels were far higher than other family members. The expression of *HMEI_Hman16* was slightly lower than the above two, but still markedly higher than other homologous paralogs (File S6), although its TPM values are largely derived from ambiguous read assignment and its quantification is therefore less reliable. The remaining 15 paralogs maintained low basal expression, with TPM values generally below 300. Pairwise Wilcoxon signed-rank tests with FDR correction (File S9) confirmed that *HMEI_Hman06* was significantly more highly expressed than 17 of 17 remaining paralogs, *HMEI_Hman15* than 16 of 17, and *HMEI_Hman16* than 15 of 17, fully consistent with the bar chart.

#### 3.4.2. Inter-Paralog Expression of *Whitmania pigra*

**Figure 7:**
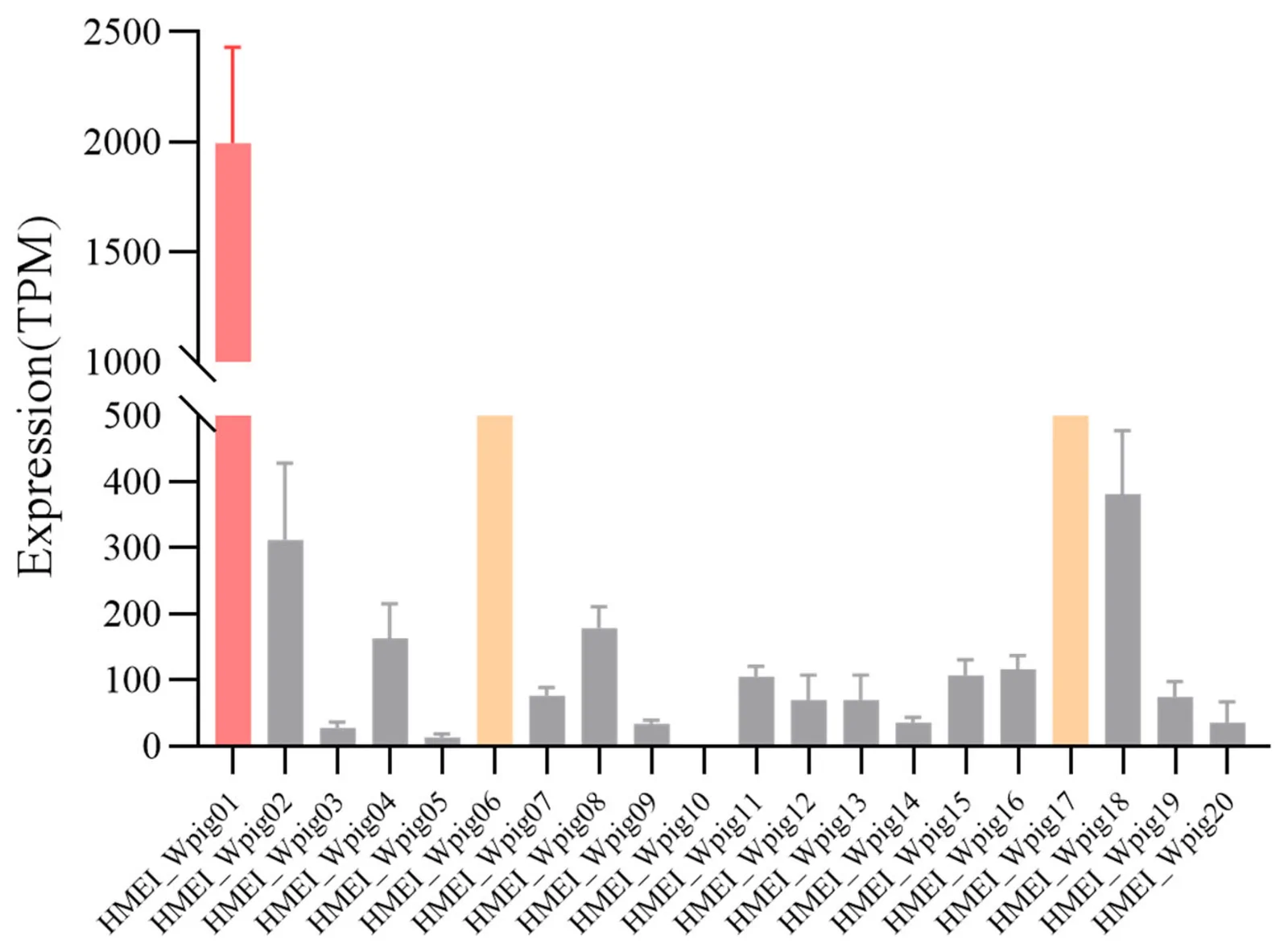
Expression levels of individual genes in the HMEI gene family of *W. pigra.* The vertical axis indicates gene expression levels; the horizontal axis indicates each HMEI gene. Bar heights correspond to mean expression values. Bars are colour-coded by mean TPM: red, mean TPM ≥ 1000; yellow, 500 ≤ mean TPM < 1000; grey, mean TPM < 500. Error bars denote the standard error across biological replicates, and a broken *y*-axis was applied to accommodate the wide range of transcriptional abundance. Data are based on *n* = 57 biological replicates.

SPSS results (Table 7) and Friedman test revealed extremely significant expression differences among the 20 HMEI of *W. pigra* (*χ^2^* = 537.591, *df* = 19, *p* < 0.001). The bar chart (Figure 7) clearly identified three highly expressed paralogs (*01*, *17*, *06*) in the HMEI gene family of *W. pigra*. *HMEI_Wpig01* showed the highest transcriptional level in the whole family, while *HMEI_Wpig17* and *HMEI_Wpig06* also maintained high expression. In addition, multiple paralogs, including *HMEI_Wpig18*, *02*, *08*, and *04*, exhibited moderate expression activity. Although most paralogs had low transcription levels, no widespread gene silencing was observed. The same post hoc analysis (File S9) confirmed that *HMEI_Wpig01* and *HMEI_Wpig17* were significantly more highly expressed than 17 of 19 remaining paralogs, and *HMEI_Wpig06* was significantly more highly expressed than 16 of the remaining 19 paralogs. But for *HMEI_Wpig06*, its TPM values were largely derived from ambiguously read assignments (File S6), making its quantification unreliable.

## 4. Discussion

Previous genomic studies identified 18 HMEI paralogs in *H. manillensis* and 20 in *W. pigra* [6,9,16,46]. The present study further compared their expression across five natural populations of each species. Although total HMEI transcript abundance did not differ significantly between species, individual paralogs contributed differently to family expression. Expression in *H. manillensis* was concentrated in a few members, particularly *HMEI_Hman06* and *HMEI_Hman15*, whereas *W. pigra* showed a broader distribution of expression among paralogs. These findings extend the previous family-level comparison [16] and show that total transcript abundance alone does not fully describe the expression differences between the two species.

The phylogenetic analysis revealed interleaved clustering of HMEI sequences from both species, together with groups containing sequences from only one species. This pattern reflects both conserved and lineage-specific components within the family. However, given the low support for some internal nodes and the inclusion of only two species in this study, we interpret these groups as descriptive sequence groups rather than established evolutionary lineages. In particular, the weak support for G3 does not establish an orthologous relationship between *HMEI_Hman06* and *HMEI_Wpig18*. Broader species sampling and genomic synteny analysis are needed to clarify these relationships.

The expression differences among HMEI paralogs suggest that family members are not transcriptionally equivalent in the sampled head tissues. Studies of duplicate genes in mammals have likewise documented divergence in expression levels and tissue preferences following duplication [55]. This provides a useful comparison, although the mechanisms responsible for HMEI expression differences remain unknown. Moreover, the presence and expression of HMEI in non-hematophagous *W. pigra* should not be interpreted solely in relation to bloodfeeding. Iwama et al. [32] identified homologues of several anticoagulant-associated proteins in non-bloodfeeding leeches and related annelids, while Babenko et al. [21] found that several known salivary components were also expressed outside salivary cells. These studies support investigating possible endogenous roles of HMEI alongside its effects on vertebrate proteases.

Population comparisons showed that variation in individual paralogs was not always reflected in total family expression. In *H. manillensis*, nine paralogs differed significantly among populations after FDR correction, although total HMEI expression did not. In *W. pigra*, population differences were detected in both total expression and 17 paralogs. These results identify population-associated expression variation, but do not distinguish genetic effects from environmental or physiological influences. Lemke et al. [56] reported changes in hirudin transcript abundance and salivary protein replenishment after feeding in *H. verbana*, demonstrating that feeding state can affect leech bioactive components. Although HMEI was not examined in that study, its findings highlight the need to standardize feeding history in future comparisons. The two-week maintenance period used here cannot establish that all sampled individuals had reached equivalent physiological states.

*HMEI_Hman06*, *HMEI_Hman15*, *HMEI_Wpig01*, and *HMEI_Wpig17* were selected as candidates for further characterization because they combined relatively high expression with supporting unique-read and sequence-divergence evidence. By contrast, the predominantly ambiguous read assignments for *HMEI_Hman16* and *HMEI_Wpig06* limit interpretation of their individual TPM estimates. Such uncertainty is a recognized problem when quantifying closely related paralogs using short-read sequencing [57]. Functional characterization is also essential: Müller et al. [17] showed that related hirudins and hirudin-like factors in *W. pigra* differ substantially in thrombin-inhibitory activity. For HMEI, Xu et al. [44] demonstrated that HMEI-A inhibits elastase and suppresses NET formation, but these activities cannot be assumed for other family members. Recombinant protein assays should first determine the inhibitory potency and protease selectivity of the selected candidates, followed by NET-formation assays where appropriate.

This study has several limitations. Transcript abundance in head tissues does not directly reflect HMEI protein levels, secretion, or biological activity, and the inclusion of only two species limits the resolution of the family’s evolutionary history. Future studies should expand taxonomic and tissue sampling and combine protein-level measurements with functional assays to clarify the expression patterns and biochemical functions of individual HMEI members. Comparing populations from different sources under standardized rearing and feeding conditions, including commercially farmed *W. pigra* and different breeding lines, would help distinguish the effects of genetic background from environmental and physiological influences on expression and assess whether these expression patterns extend to medicinal production populations.

## 5. Conclusions

The two medicinal leech species differed in the distribution of HMEI expression among paralogs, despite the absence of a significant interspecies difference in total family transcript abundance. Population-associated variation further showed that family totals do not capture all changes in individual members. *HMEI_Hman06*, *HMEI_Hman15*, *HMEI_Wpig01*, and *HMEI_Wpig17* were identified as expression-based candidates for biochemical characterization. These results extend the comparison of HMEI from family-level abundance to individual paralogs across natural populations and provide a basis for testing their inhibitory activities and biological roles.

## Figures and Tables

**Table 1:** Sampling localities and sample sizes of the two leech species.

Species	Locality	City, Province	Longitude (°E)	Latitude (°N)	Sample Size
*H. manillensis*	GXGG	Guigang, Guangxi	109.600	23.100	10
*H. manillensis*	GXQZ	Qinzhou, Guangxi	108.650	21.983	10
*H. manillensis*	GDMM	Maoming, Guangdong	110.917	21.650	10
*H. manillensis*	HNDA	Ding’an, Hainan	110.450	19.767	12
*H. manillensis*	YNHH	Honghe, Yunnan	103.383	23.383	12
*W. pigra*	JXJA	Ji’an, Jiangxi	114.983	27.067	10
*W. pigra*	HBZX	Zhongxiang, Hubei	112.633	31.117	12
*W. pigra*	TJBD	Baodi, Tianjin	117.500	39.717	12
*W. pigra*	SCYB	Yibin, Sichuan	104.600	28.750	11
*W. pigra*	HBWH	Wuhan, Hubei	114.300	30.550	12

Geographical coordinates are expressed in decimal degrees. *H. manillensis*: *n* = 54; *W. pigra*: *n* = 57; total: *n* = 111.

**Table 2:** Comparison of TPM values across descriptive sequence groups of *H. manillensis* and *W. pigra*.

Descriptive Sequence Groups			TPM (Mean ± SE)		Mann–Whitney *U* Test	BH-FDR
Group	*H. manillensis*	*W. pigra*	*H. manillensis*	*W. pigra*	*p*	*q*
G1	*HMEI_Hman08* *HMEI_Hman11*	*HMEI_Wpig01* *HMEI_Wpig02* *HMEI_Wpig04* *HMEI_Wpig05* *HMEI_Wpig06* *HMEI_Wpig08* *HMEI_Wpig09* *HMEI_Wpig11* *HMEI_Wpig12* *HMEI_Wpig13* *HMEI_Wpig14* *HMEI_Wpig15* *HMEI_Wpig17*	0.31 ± 0.08	352.11 ± 46.42	<0.001	3.4 × 10^−19^
G2	*HMEI_Hman01*	—	15.70 ± 9.70	—	—	—
G3	*HMEI_Hman06*	*HMEI_Wpig18*	**2573.06 ± 330.67**	381.35 ± 96.34	<0.001	4.8 × 10^−12^
G4	*HMEI_Hman09* *HMEI_Hman12* *HMEI_Hman18*	*HMEI_Wpig03* *HMEI_Wpig16*	80.01 ± 10.36	71.74 ± 15.01	0.229	0.229
G5	*HMEI_Hman14* *HMEI_Hman15* *HMEI_Hman16* *HMEI_Hman17*	*HMEI_Wpig07*	699.95 ± 81.68	76.02 ± 12.81	<0.001	1.8 × 10^−14^
G6	—	*HMEI_Wpig10*	—	0.14 ± 0.13	—	—
G7	—	*HMEI_Wpig19* *HMEI_Wpig20*	—	54.92 ± 19.24	—	—
G8	*HMEI_Hman03* *HMEI_Hman04* *HMEI_Hman05* *HMEI_Hman07* *HMEI_Hman10* *HMEI_Hman13*	—	1.40 ± 1.08	—	—	—
G9	*HMEI_Hman02*	—	0.81 ± 0.12	—	—	—

TPM, transcripts per million; SE, standard error. Genes with TPM > 1000 are shown in bold. —, not applicable; The group value of each individual was calculated as the per-individual mean of the member paralogs’ TPM values. The Mann–Whitney *U* test was performed on these per-individual values (individual leeches as the independent biological units) for the four groups containing paralogs from both species (G1, G3, G4 and G5); raw *p* values and Benjamini–Hochberg adjusted values (q), calculated from the unrounded *p* values, are both reported. Exact *p* values are provided in File S9.

**Table 3:** Comparison of TPM values of representative paralogs from each descriptive sequence group of *H. manillensis* and *W. pigra*.

Representative Gene			TPM (Mean ± SE)	
Group	*H. manillensis*	*W. pigra*	*H. manillensis*	*W. pigra*
G1	*HMEI_Hman08*	*HMEI_Wpig01*	0.31 ± 0.10	**1994.65 ± 436.51**
G2	*HMEI_Hman01*	15.70 ± 9.70	—	—
G3	*HMEI_Hman06*	*HMEI_Wpig18*	**2573.06 ± 330.67**	381.36 ± 96.34
G4	*HMEI_Hman09*	*HMEI_Wpig16*	189.25 ± 27.94	116.22 ± 21.37
G5	*HMEI_Hman15*	*HMEI_Wpig07*	**1805.21 ± 264.23**	76.02 ± 12.81
G6	—	*HMEI_Wpig10*	—	0.14 ± 0.13
G7	—	*HMEI_Wpig19*	—	74.01 ± 23.85
G8	*HMEI_Hman13*	—	2.59 ± 2.43	—
G9	*HMEI_Hman02*	—	0.81 ± 0.12	—

TPM, transcripts per million; SE, standard error. Genes with TPM > 1000 are shown in bold.

**Table 4:** Comparison of TPM values of HMEI across different populations of *H. manillensis*.

Genes	GXGG	GXQZ	GDMM	HNDA	YNHH
*HMEI_Hman01*	8.44 ± 6.95 (6)	11.84 ± 11.81 (3)	4.08 ± 2.31 (6)	50.36 ± 41.97 (9)	0
*HMEI_Hman02*	0.40 ± 0.20 (8)	1.45 ± 0.26 (10)	0.14 ± 0.05 (5)	0.92 ± 0.28 (11)	1.07 ± 0.23 (11)
*HMEI_Hman03*	0.03 ± 0.01 (4)	8.93 ± 6.45 (3)	0.01 ± 0.01 (1)	0.01 ± 0.01 (1)	0.02 ± 0.01 (3)
*HMEI_Hman04*	0.03 ± 0.01 (4)	8.93 ± 6.45 (3)	0.01 ± 0.01 (1)	0.01 ± 0.01 (1)	0.02 ± 0.01 (3)
*HMEI_Hman05*	0.03 ± 0.01 (4)	8.93 ± 6.45 (3)	0.01 ± 0.01 (1)	0.01 ± 0.01 (1)	0.02 ± 0.01 (3)
*HMEI_Hman06*	**1951.63 ± 586.72 (10)**	**2546.27 ± 797.66 (10)**	**2779.04 ± 561.60 (10)**	**3543.13 ± 1042.55 (12)**	**1971.53 ± 499.34 (12)**
*HMEI_Hman07*	0	1.86 ± 1.40 (2)	0	0	0
*HMEI_Hman08*	0.39 ± 0.15 (7)	0.08 ± 0.04 (4)	0.83 ± 0.40 (8)	0.32 ± 0.24 (6)	0.01 ± 0.01 (1)
*HMEI_Hman09*	142.48 ± 23.09 (10)	75.26 ± 29.92 (10)	309.97 ± 75.42 (10)	383.81 ± 62.98 (12)	28.08 ± 7.20 (11)
*HMEI_Hman10*	0	2.48 ± 2.29 (2)	0	0.01 ± 0.01 (1)	0
*HMEI_Hman11*	0.14 ± 0.06 (6)	0.10 ± 0.05 (5)	0.88 ± 0.28 (8)	0.43 ± 0.18 (7)	0.01 ± 0.01 (1)
*HMEI_Hman12*	10.74 ± 3.61 (10)	18.22 ± 13.35 (9)	29.20 ± 14.01 (8)	36.08 ± 15.54 (12)	5.83 ± 2.34 (8)
*HMEI_Hman13*	0.07 ± 0.06 (2)	13.93 ± 13.06 (2)	0	0	0
*HMEI_Hman14*	45.62 ± 9.53 (10)	101.71 ± 31.55 (10)	45.23 ± 8.24 (10)	149.93 ± 31.75 (12)	173.46 ± 82.55 (12)
*HMEI_Hman15*	872.53 ± 223.31 (10)	**1228.21 ± 477.13 (10)**	**1422.02 ± 344.93 (10)**	**3896.09 ± 778.65 (12)**	**1291.72 ± 336.83 (12)**
*HMEI_Hman16*	441.74 ± 179.23 (10)	**1830.18 ± 457.72 (10)**	644.68 ± 169.58 (10)	845.64 ± 258.27 (12)	191.58 ± 126.38 (12)
*HMEI_Hman17*	30.60 ± 6.40 (10)	14.41 ± 8.77 (8)	91.52 ± 24.36 (10)	202.81 ± 29.12 (12)	207.45 ± 51.10 (11)
*HMEI_Hman18*	21.52 ± 6.45 (9)	30.39 ± 12.06 (10)	56.09 ± 17.12 (10)	38.10 ± 6.45 (11)	10.08 ± 3.11 (10)
mean	195.91 ± 58.10	327.96 ± 104.25	299.09 ± 67.69	508.20 ± 126.00	215.60 ± 61.62

GXGG, Guigang, Guangxi; GXQZ, Qinzhou, Guangxi; GDMM, Maoming, Guangdong; HNDA, Ding’an, Hainan; YNHH, Honghe, Yunnan. Genes with TPM > 1000 are shown in bold. Values are mean ± SE (*n*); *n* = number of individuals with TPM > 0. The “mean” row represents the average of the per-gene mean TPM values within each population. Cells marked ‘0’ indicate that no expression of this paralog was detected in any individual of the corresponding population (TPM = 0 in all replicates); therefore, no mean ± SE is reported for these cells. In cells with a single detected individual (*n* = 1), the mean equals the SE; values below the two-decimal display precision are shown as 0.01 to indicate detectable expression distinct from zero.

**Table 5:** Comparison of TPM values of HMEI across different populations of *W. pigra*.

Genes	JXJA	HBZX	TJBD	SCYB	HBWH
*HMEI_Wpig01*	**6496.50 ± 1021.25 (10)**	**1538.71 ± 961.65 (12)**	401.46 ± 355.79 (12)	**1372.41 ± 729.07 (11)**	862.65 ± 684.43 (12)
*HMEI_Wpig02*	8.38 ± 1.47 (10)	7.80 ± 3.16 (12)	**1438.83 ± 424.87 (12)**	14.32 ± 3.44 (11)	16.04 ± 9.34 (12)
*HMEI_Wpig03*	13.41 ± 8.46 (9)	7.36 ± 5.29 (5)	22.54 ± 7.97 (12)	34.62 ± 8.23 (11)	56.70 ± 41.77 (9)
*HMEI_Wpig04*	7.29 ± 1.53 (10)	52.96 ± 31.00 (11)	601.33 ± 202.63 (12)	30.85 ± 8.10 (11)	86.62 ± 56.72 (11)
*HMEI_Wpig05*	0.10 ± 0.10 (1)	0.66 ± 0.46 (3)	1.21 ± 0.56 (9)	3.40 ± 1.59 (7)	56.00 ± 22.49 (8)
*HMEI_Wpig06*	163.85 ± 39.04 (10)	220.03 ± 167.25 (12)	492.85 ± 88.70 (12)	681.90 ± 127.48 (11)	**1154.95 ± 552.14 (12)**
*HMEI_Wpig07*	219.77 ± 37.84 (10)	81.89 ± 22.07 (11)	17.57 ± 5.04 (12)	30.21 ± 11.00 (11)	50.78 ± 16.28 (12)
*HMEI_Wpig08*	210.67 ± 46.65 (10)	141.15 ± 56.08 (12)	90.18 ± 46.69 (12)	366.94 ± 122.55 (11)	105.13 ± 46.22 (12)
*HMEI_Wpig09*	35.97 ± 7.35 (10)	8.15 ± 2.58 (11)	61.96 ± 11.26 (12)	65.09 ± 10.62 (11)	2.26 ± 0.49 (9)
*HMEI_Wpig10*	0	0.65 ± 0.63 (2)	0.01 ± 0.01 (1)	0	0
*HMEI_Wpig11*	35.15 ± 6.88 (10)	56.32 ± 13.75 (12)	89.27 ± 17.18 (12)	267.31 ± 43.89 (11)	79.68 ± 32.97 (12)
*HMEI_Wpig12*	2.96 ± 0.99 (10)	7.01 ± 6.37 (8)	0.21 ± 0.08 (7)	0.61 ± 0.17 (10)	322.56 ± 165.50 (12)
*HMEI_Wpig13*	1.89 ± 1.05 (7)	6.68 ± 6.40 (7)	0.50 ± 0.14 (11)	0.83 ± 0.23 (10)	322.12 ± 165.45 (10)
*HMEI_Wpig14*	15.06 ± 5.18 (10)	11.83 ± 4.56 (12)	35.59 ± 7.49 (12)	20.31 ± 7.72 (11)	89.89 ± 32.24 (12)
*HMEI_Wpig15*	29.07 ± 7.06 (10)	75.47 ± 22.44 (11)	72.95 ± 21.60 (12)	34.62 ± 6.22 (11)	302.90 ± 95.13 (12)
*HMEI_Wpig16*	33.20 ± 22.02 (9)	90.85 ± 33.04 (11)	92.92 ± 31.07 (12)	144.99 ± 30.03 (11)	207.68 ± 80.26 (12)
*HMEI_Wpig17*	**1554.79 ± 481.95 (10)**	314.04 ± 174.43 (10)	806.89 ± 552.08 (12)	**1846.02 ± 742.43 (11)**	360.45 ± 115.55 (12)
*HMEI_Wpig18*	627.48 ± 137.83 (10)	338.93 ± 219.91 (12)	368.79 ± 320.63 (12)	464.49 ± 220.94 (11)	155.04 ± 98.26 (12)
*HMEI_Wpig19*	2.31 ± 1.83 (3)	65.41 ± 41.98 (8)	55.11 ± 32.34 (12)	198.88 ± 100.32 (9)	46.78 ± 27.54 (11)
*HMEI_Wpig20*	198.11 ± 175.52 (2)	5.10 ± 4.63 (2)	0	0	0
mean	482.80 ± 100.20	151.55 ± 88.88	232.51 ± 106.31	278.89 ± 108.70	213.91 ± 112.14

JXJA, Ji’an, Jiangxi; HBZX, Zhongxiang, Hubei; TJBD, Baodi, Tianjin; SCYB, Yibin, Sichuan; HBWH, Wuhan, Hubei. Genes with TPM > 1000 are shown in bold. Values are mean ± SE (*n*); *n* = number of individuals with TPM > 0. The “mean” row represents the average of the per-gene mean TPM values within each population. Cells marked ‘0’ indicate that no expression of this paralog was detected in any individual of the corresponding population (TPM = 0 in all replicates); therefore, no mean ± SE is reported for these cells.

**Table 6:** TPM comparison of individual HMEI in *H. manillensis*.

Genes	Mean ± SE
*HMEI_Hman01*	15.70 ± 9.70
*HMEI_Hman02*	0.81 ± 0.12
*HMEI_Hman03*	1.67 ± 1.24
*HMEI_Hman04*	1.67 ± 1.24
*HMEI_Hman05*	1.66 ± 1.24
*HMEI_Hman06*	**2573.10 ± 330.67**
*HMEI_Hman07*	0.34 ± 0.27
*HMEI_Hman08*	0.31 ± 0.10
*HMEI_Hman09*	189.25 ± 27.94
*HMEI_Hman10*	0.46 ± 0.43
*HMEI_Hman11*	0.31 ± 0.08
*HMEI_Hman12*	20.08 ± 5.11
*HMEI_Hman13*	2.59 ± 2.43
*HMEI_Hman14*	107.52 ± 21.21
*HMEI_Hman15*	**1805.20 ± 264.23**
*HMEI_Hman16*	**770.61 ± 134.54**
*HMEI_Hman17*	116.45 ± 17.67
*HMEI_Hman18*	30.71 ± 4.69

TPM, transcripts per million; SE, standard error. Genes with TPM > 500 are shown in bold. Data are presented as mean ± SE (*n* = 54 biological replicates).

**Table 7:** TPM comparison of individual HMEI in *W. pigra*.

Genes	Mean ± SE
*HMEI_Wpig01*	**1994.70 ± 436.51**
*HMEI_Wpig02*	312.16 ± 116.25
*HMEI_Wpig03*	27.26 ± 9.27
*HMEI_Wpig04*	163.21 ± 52.90
*HMEI_Wpig05*	12.86 ± 5.47
*HMEI_Wpig06*	**553.57 ± 130.45**
*HMEI_Wpig07*	76.01 ± 12.81
*HMEI_Wpig08*	178.60 ± 32.68
*HMEI_Wpig09*	34.11 ± 4.85
*HMEI_Wpig10*	0.14 ± 0.13
*HMEI_Wpig11*	105.18 ± 15.84
*HMEI_Wpig12*	70.06 ± 37.92
*HMEI_Wpig13*	69.82 ± 37.91
*HMEI_Wpig14*	35.47 ± 8.02
*HMEI_Wpig15*	106.80 ± 24.63
*HMEI_Wpig16*	116.22 ± 21.37
*HMEI_Wpig17*	**940.89 ± 216.27**
*HMEI_Wpig18*	381.36 ± 96.34
*HMEI_Wpig19*	74.00 ± 23.85
*HMEI_Wpig20*	35.83 ± 31.14

TPM, transcripts per million; SE, standard error. Genes with TPM > 500 are shown in bold. Data are presented as mean ± SE (*n* = 57 biological replicates).

## Data Availability

Data are contained within the article and Supplementary Files. All raw RNA-seq data have been deposited in the Genome Sequence Archive (GSA) at the National Genomics Data Center (NGDC), Beijing Institute of Genomics, Chinese Academy of Sciences, under BioProject accession numbers PRJCA046291 (*W. pigra*, TJBD population) and PRJCA076553 (all other samples).

## Supplementary Materials

The following supporting information can be downloaded at: https://www.mdpi.com/article/10.3390/biology15211973/s1, File S1: HMEI CDSs obtained in *H. manillensis* and *W. pigra* samples; File S2: Open reading frame analysis of HMEI paralogs in *H. manillensis* and *W. pigra*; File S3: Amino acid sequences of all HMEI paralogs from *H. manillensis* and *W. pigra*; File S4: Multiple nucleotide sequence alignment of HMEI coding sequences used for phylogenetic analysis; File S5: TPM values of HMEI genes of *H. manillensis* and *W. pigra* samples; File S6: Unique and ambiguous read counts of all HMEI paralogs in *H. manillensis* and *W. pigra* (from Salmon aux_info/ambig_info.tsv); File S7: Pairwise nucleotide distance matrix of HMEI paralogs, with the minimum pairwise distance of each core paralog to its closest paralog reported in Sheet 2; File S8: Genomic coordinates of the identical HMEI paralogs (*HMEI_Hman03/04/05* and *HMEI_Wpig12/13*) in GFF format; File S9: Kruskal–Wallis *H* test results and Benjamini–Hochberg FDR correction (with Wilcoxon post hoc comparisons) for within-species inter-paralog and inter-population expression analyses.

## Abbreviations

The following abbreviations are used in this manuscript:

bp	base pair
BH	Benjamini–Hochberg
HMEI	*Hirudinaria manillensis* elastase inhibitor
LDTI	leech-derived tryptase inhibitor
NE	neutrophil elastase
NETs	neutrophil extracellular traps
NF-κB	nuclear factor kappa-B
NLRP3	NOD-like receptor protein 3
RNA-Seq	RNA sequencing
SE	standard error
SPSS	Statistical Package for the Social Sciences
TPM	transcripts per million

## References

[1] Sket B., Trontelj P. (2008). Global Diversity of Leeches (Hirudinea) in Freshwater. Hydrobiologia.

[2] Yang T., Editorial Committee of Fauna Sinica, Academia Sinica (1996). Fauna Sinica, Invertebrata Vol. 5: Annelida, Hirudinea.

[3] Kalatehjari P., Wolf R., Jedlitschky G., Tolksdorf C., Rauch B.H., Müller C. (2026). The Great Diversity: Monomeric and Oligomeric Hirudins, Hirudin-like Factors and Decorsins in the Asian Medicinal Leeches *Hirudo nipponia* and *Hirudo tianjinensis*. Parasitol. Res..

[4] Ma C., Li X., Chen H. (2021). Research Progress in the Use of Leeches for Medical Purposes. Tradit. Med. Res..

[5] Jiang Q., Wang L., Liu Q., Yang C., Zhang Y. (2022). Research Progress on Processing History Evolution, Chemical Constituents and Pharmacological Effects of Hirudo. China J. Chin. Mater. Med..

[6] Liu Z., Zhao F., Huang Z., Hu Q., Meng R., Lin Y., Qi J., Lin G. (2023). Revisiting the Asian Buffalo Leech (*Hirudinaria manillensis*) Genome: Focus on Antithrombotic Genes and Their Corresponding Proteins. Genes.

[7] Ye T., Zhao F., Xiao M., Yin J., Ai R., Tang L., Liu Z., Huang Z., Lin G. (2025). Comparative Study of Lefaxin Family in Two Asian Leeches: *Hirudinaria manillensis* and *Whitmania pigra*. Biology.

[8] Tessler M., de Carle D., Voiklis M.L., Gresham O.A., Neumann J.S., Cios S., Siddall M.E. (2018). Worms That Suck: Phylogenetic Analysis of Hirudinea Solidifies the Position of Acanthobdellida and Necessitates the Dissolution of Rhynchobdellida. Mol. Phylogenet. Evol..

[9] Lin G., Yin J., Zhang W., Huang Z., Liu Z., Chen H., Tang L., Zhao F. (2025). Population Genetics of the Asian Buffalo Leech (*Hirudinaria manillensis*) in Southern China Based on Mitochondrial Protein-Coding Genes. Biology.

[10] Abdualkader A.M., Ghawi A.M., Alaama M., Awang M., Merzouk A. (2013). Leech Therapeutic Applications. Indian J. Pharm. Sci..

[11] Yu Y., Tang L., Xiao M., Yin J., Ye T., Sun R., Ai R., Zhao F., Huang Z., Lin G. (2025). Genetic Variation and Gene Expression of the Antimicrobial Peptide Macins in Asian Buffalo Leech (*Hirudinaria manillensis*). Biology.

[12] Zhang B., Wang B., Gong Y., Yu X., Lü J. (2012). Anticoagulant Active Substances Extraction and Anti-Thrombin Activity Analysis of Several Species of Leeches. Acta Sci. Nat. Univ. Sunyatseni.

[13] Guangxi Zhuang Autonomous Region Drug Administration (2011). Guangxi Zhuang Autonomous Region Medicine Quality Standards.

[14] Yunnan Provincial Drug Administration (2013). Yunnan Chinese Medicinal Materials Standard.

[15] Tan E. (2007). Zoogeoraphy of Hirudinidae in China. Acta Sci. Nat. Univ. Sunyatseni.

[16] Liu Z., Zhao F., Huang Z., He B., Liu K., Shi F., Zhao Z., Lin G. (2024). A Chromosome-Level Genome Assembly of the Non-Hematophagous Leech *Whitmania pigra* (Whitman 1884): Identification and Expression Analysis of Antithrombotic Genes. Genes.

[17] Müller C., Wang Z., Hamann M., Sponholz D., Hildebrandt J.-P. (2022). Life without Blood: Molecular and Functional Analysis of Hirudins and Hirudin-like Factors of the Asian Non-Hematophagous Leech *Whitmania pigra*. J. Thromb. Haemost..

[18] National Pharmacopoeia Committee of China (2025). Chinese Pharmacopoeia 2025.

[19] Junren C., Xiaofang X., Huiqiong Z., Gangmin L., Yanpeng Y., Xiaoyu C., Yuqing G., Yanan L., Yue Z., Fu P. (2021). Pharmacological Activities and Mechanisms of Hirudin and Its Derivatives—A Review. Front. Pharmacol..

[20] Greinacher A., Warkentin T.E. (2008). The Direct Thrombin Inhibitor Hirudin. Thromb. Haemost..

[21] Babenko V.V., Podgorny O.V., Manuvera V.A., Kasianov A.S., Manolov A.I., Grafskaia E.N., Shirokov D.A., Kurdyumov A.S., Vinogradov D.V., Nikitina A.S. (2020). Draft Genome Sequences of *Hirudo medicinalis* and Salivary Transcriptome of Three Closely Related Medicinal Leeches. BMC Genom..

[22] Kvist S., Manzano-Marín A., de Carle D., Trontelj P., Siddall M.E. (2020). Draft Genome of the European Medicinal Leech *Hirudo medicinalis* (Annelida, Clitellata, Hirudiniformes) with Emphasis on Anticoagulants. Sci. Rep..

[23] Shan H., Ren K., Liu J., Rehman S.U., Yan X., Ma X., Zheng Y., Feng T., Wang X., Li Z. (2022). Comprehensive Transcriptome Sequencing Analysis of *Hirudinaria manillensis* in Different Growth Periods. Front. Physiol..

[24] Sommerhoff C.P., Söllner C., Mentele R., Piechottka G.P., Auerswald E.A., Fritz H. (1994). A Kazal-Type Inhibitor of Human Mast Cell Tryptase: Isolation from the Medical Leech *Hirudo medicinalis*, Characterization, and Sequence Analysis. Biol. Chem. Hoppe-Seyler.

[25] Nutt E., Gasic T., Rodkey J., Gasic G.J., Jacobs J.W., Friedman P.A., Simpson E. (1988). The Amino Acid Sequence of Antistasin. A Potent Inhibitor of Factor Xa Reveals a Repeated Internal Structure. J. Biol. Chem..

[26] Baskova I.P., Zavalova L.L. (2001). Proteinase Inhibitors from the Medicinal Leech *Hirudo medicinalis*. Biochemistry.

[27] Faria F., Kelen E.M.A., Sampaio C.A.M., Bon C., Duval N., Chudzinski-Tavassi A.M. (1999). A New Factor Xa Inhibitor (Lefaxin) from the *Haementeria depressa* Leech. Thromb. Haemost..

[28] Sun R., Ai R., Yin J., Cheng J., Huang Z., Tang L., Liu Z., Zeng Q., Zhao F., Lin G. (2025). Comparative Study of Hirustasin Superfamily Gene Expression in Two Medicinal Leeches, *Hirudinaria manillensis* and *Whitmania pigra*. Genes.

[29] Yin J., Liu Z., Yu Y., Wang A., Huang Z., Tang L., Zhao F., Lin G. (2025). Comparative Study of Hirudins and Encoding Genes in *Hirudo nipponia* and *Hirudo tianjinensis*. Biology.

[30] Lin Y., Zhao F., Fan S., Yang D., Tong X., Tang L., Kong D., Lin G., Liu Z. (2025). Comparative Genomics Suggests Extensive Antithrombotic Gene Expansion in *Haemadipsa yanyuanensis*. BMC Genom..

[31] Zheng J., Wang X., Feng T., Rehman S.U., Yan X., Shan H., Ma X., Zhou W., Xu W., Lu L. (2022). Molecular Mechanisms Underlying Hematophagia Revealed by Comparative Analyses of Leech Genomes. GigaScience.

[32] Iwama R.E., Tessler M., Kvist S. (2022). Leech Anticoagulants Are Ancestral and Likely to Be Multifunctional. Zool. J. Linn. Soc..

[33] Zhao F., Huang Z., Tang L., Zhang W., Liu Z., Lin G. (2025). Comparative Genomics of Three Non-Hematophagous Leeches (*Whitmania* spp.) with Emphasis on Antithrombotic Biomolecules. Front. Genet..

[34] He M.X., Li Z. (2019). Y Extraction and SDS-PAGE Electrophoresis of Anticoagulant Components from *Poecilobdella manillensis* Lesson in Hainan. J. South China Univ. Trop. Agric..

[35] Sun Y., Wang Y., Liu J., Al E. (2021). Research Progress in the Analysis of Anticoagulant Active Substances in Leeches and the Extraction and Purification of Hirudin. Acta Neuropharmacol..

[36] Raghavi S., Deva darshini B., Saravanan K.M., Anbarasu K. (2026). Molecular Insights into Leech-Derived Bioactive Compounds: Biochemical Mechanisms and Therapeutic Potential. Int. J. Mol. Sci..

[37] Zheng H., Deng X., Li T., Xia G., Yang H., Peng J., Shen Y. (2026). Mining Anticoagulant Peptides from *Poecilobdella manillensis* by Peptidomics Analysis. Nat. Prod. Bioprospect..

[38] Xu H., Zhang D., Lu J., Cui Y., Wu L., Chen Z., Jin Z., Lü Z., Xu P., Zhang Y. (2023). Mechanism of Hirudo in Treatment of Stroke: A Review. Chin. J. Exp. Tradit. Med. Formulae.

[39] Low A., Mak E., Rowe J.B., Markus H.S., O’Brien J.T. (2019). Inflammation and Cerebral Small Vessel Disease: A Systematic Review. Ageing Res. Rev..

[40] Pajares M., I Rojo A., Manda G., Boscá L., Cuadrado A. (2020). Inflammation in Parkinson’s Disease: Mechanisms and Therapeutic Implications. Cells.

[41] Toenders Y.J., Laskaris L., Davey C.G., Berk M., Milaneschi Y., Lamers F., Penninx B.W.J.H., Schmaal L. (2022). Inflammation and Depression in Young People: A Systematic Review and Proposed Inflammatory Pathways. Mol. Psychiatry.

[42] Zhou Y., Xu Z., Liu Z. (2022). Impact of Neutrophil Extracellular Traps on Thrombosis Formation: New Findings and Future Perspective. Front. Cell. Infect. Microbiol..

[43] Papayannopoulos V. (2018). Neutrophil Extracellular Traps in Immunity and Disease. Nat. Rev. Immunol..

[44] Xu K.-H., Zhou M., Wu F.-L., Tang X.-P., Lu Q.-M., Lai R., Long C.-B. (2021). Identification and Characterization of a Novel Elastase Inhibitor from *Hirudinaria manillensis*. Chin. J. Nat. Med..

[45] Pham C.T.N. (2006). Neutrophil Serine Proteases: Specific Regulators of Inflammation. Nat. Rev. Immunol..

[46] Pan Y., Zhou P., Luo Y., Yang M., Chen H., Liu Z., Zhao F., Lin G., Tang L. (2026). Population Genetics of the Medicinal Leech *Whitmania pigra* Based on Mitochondrial Genes. Genes.

[47] Chen S., Zhou Y., Chen Y., Gu J. (2018). Fastp: An Ultra-Fast All-in-One FASTQ Preprocessor. Bioinformatics.

[48] Tamura K., Stecher G., Kumar S. (2021). MEGA11: Molecular Evolutionary Genetics Analysis Version 11. Mol. Biol. Evol..

[49] Patro R., Duggal G., Love M.I., Irizarry R.A., Kingsford C. (2017). Salmon Provides Fast and Bias-Aware Quantification of Transcript Expression. Nat. Methods.

[50] IBM Corp (2019). IBM SPSS Statistics for Windows, Version 26.0.

[51] OriginLab Corporation (2020). Origin, Version 2021.

[52] Han S., Liu M., Zhang X., Piao J., Jia L., Liu J., Xia H. (2026). Genome-Wide Identification and Expression Analysis of DUF568 Gene Family in Maize (*Zea mays*) under Abiotic Stresses. Agronomy.

[53] Hu H., He L., Chen R., Jing Z., Wan H., Xiao Z., Cheng H., Wu C., Liu X. (2026). Genome-Wide Identification and Multi-Dimensional Functional Characterization of the SIR2 Family in *Brassica napus* L. PLoS ONE.

[54] GraphPad Software (2023). GraphPad Prism, Version 10.

[55] Guschanski K., Warnefors M., Kaessmann H. (2017). The Evolution of Duplicate Gene Expression in Mammalian Organs. Genome Res..

[56] Lemke S., Müller C., Hildebrandt J.-P. (2016). Be Ready at Any Time: Postprandial Synthesis of Salivary Proteins in Salivary Gland Cells of the Haematophagous Leech *Hirudo verbana*. J. Exp. Biol..

[57] Li B., Ruotti V., Stewart R.M., Thomson J.A., Dewey C.N. (2010). RNA-Seq Gene Expression Estimation with Read Mapping Uncertainty. Bioinformatics.

